# Polymer-Magnetic Semiconductor Nanocomposites for Industrial Electronic Applications

**DOI:** 10.3390/polym14122467

**Published:** 2022-06-17

**Authors:** David Romero-Fierro, Moises Bustamante-Torres, Francisco Bravo-Plascencia, Héctor Magaña, Emilio Bucio

**Affiliations:** 1 Departamento de Química de Radiaciones y Radioquímica, Instituto de Ciencias Nucleares, Universidad Nacional Autónoma de México, Circuito Exterior, Ciudad Universitaria, Mexico City 04510, Mexico; moisesbustamante819@gmail.com; 2 Centro Conjunto de Investigación en Química Sustentable UAEM-UNAM, Toluca 50200, Mexico; alexanderbravo74@hotmail.es; 3 Instituto de Química, Universidad Nacional Autónoma de México, Circuito Exterior, Ciudad Universitaria, Coyoacan 04510, Mexico; 4 Facultad de Ciencias Químicas e Ingeniería, Universidad Autónoma de Baja California, Calzada Universidad 14418, Parque Industrial Internacional Tijuana, Tijuana 22390, Mexico; hector.magana@uabc.edu.mx

**Keywords:** polymer nanocomposite, reinforcement, magnetic, semiconductor

## Abstract

Nanocomposite materials have acquired great importance, as have similar composite materials on a macroscopic scale, because the reinforcement complements the defects in the properties of the matrix, thus obtaining materials with better mechanical, thermal, and electrical properties, among others. At the same time, the importance and research of polymeric nanocomposites reinforced with nanoparticles of various types have grown. Among those that have stood out the most in the electronics industry are polymeric matrices reinforced with nanoparticles that present dual behavior, that is, both magnetic and semiconductor. This property has been very well used in developing electronic devices such as televisions, computers, and smartphones, which are part of everyday life. In this sense, this review presents a compilation of the synthetic methods to produce polymer nanocomposites with dual magnetic and semiconductor behavior and their potential applications within electronic fields and new relevant trends.

## 1. Introduction

There is no doubt that magnetism is historically linked to the development of humanity. Since ancient times, magnetic materials and the phenomena related to the impulses to materialize novel ideas in various fields of application have played a role of great importance in the development of science and technology [1]. Nowadays, magnetic materials are involved in all aspects of daily human activities because they are used in a large list of optoelectronic-magnetic applications, for instance, data storing, data reading, electromagnets, transformers, motors, generators, actuators, microphones, relays, inductors, microwave isolators, antenna rods, and phase shifters [2]. Additionally, magnetic materials are used for magnetic resonance imaging, sensors, radiation sources, bioremediation, that is, for mineral separation and metal separation [3], biomedicine [4], and even in energies field [5].

In the last years, scientists have shown a strong interest in nanostructured materials because they show unusual and improved chemical and physical properties compared with their bulk counterparts [6]. This unconventional behavior shown by nanomaterial appears when they reach the quantum size regime; their size is comparable with the de Broglie wavelengths of exciton [7]. However, the factor responsible for the unique properties of nanomaterials such as nanoparticles, nanowires, and quantum dots, is the increasing predominance of the proportion of atoms present on the material surface with respect to the number of atoms belonging to the interior of the bulk as the size of the systems decreases [8,9,10]. Particularly, in the case of magnetic nanoparticles (MNPs), there is a significant change in the properties such as Curie (T_C_) or Neel (T_N_) temperatures and hysteresis behavior because, on the nanoscale, each atom will suffer higher magnetization than in bulk and anisotropy of magnetization caused by the smallness of domain which, in ferromagnetic nanoparticles, will produce superparamagnetic properties [7,11]. 

The tunable properties of magnetic nanoparticles allow their use in several applications, for instance, biomedicine [12], environmental remediation [13], catalysis and imaging [14,15], and others. However, MNPs tend to lose their colloidal stability and generate agglomerates to reduce their surface energy. To assure the colloidal stability of MNPs, some approaches have been proved, including mechanical dispersion, an increase of viscosity of the medium, and modifying the surface with macromolecules such as polymers [16]. In that context, imbibing MNPs as reinforcement phase into a polymeric matrix to generate a stable nanocomposite with fashionable properties and with a promising practical application have been highly explored [17].

Conducting polymer and hydrogel-based matrices have special importance because these types of organic macromolecules can be easily synthesized, are highly tunable and versatile materials [18], and provide nanocomposites with a semiconductor behavior that allows them to be used for optoelectronics [19]. In that way, joining MNPs into a polymer matrix allows obtaining a nanocomposite that is sensitive to exposure to magnetic fields with improved conducting and optical properties that widely expand that potential application in fields of science and industry. Therefore, this document pretends to be a comprehensive review of polymer–magnetic semiconductor nanocomposites focused mainly on the new synthetic methods and current trends in reported industrial applications. 

## 2. Fundamentals

### 2.1. Composites

Composite materials emerged in the middle of the 20th century as a promising class of engineering materials providing new prospects for modern technology [20]. Composite materials combine at least two materials, often with different properties, to get unique characteristics in which the strength-to-weight ratio is high and there is significant flexibility in the design [21]. These compounds can be selected to achieve unusual stiffness, strength, weight, high-temperature performance, corrosion resistance, toughness, or conductivity. In all composite materials, two components are distinguished: the matrix, which is the component that appears as a continuous phase and acts as a binder, and the reinforcement or filler, which is the discontinuous phase. Generally, it is said that material is composite when it meets the requirements of being made up of two or more physically different and mechanically separable components [22]. It has several chemically different phases, insoluble among themselves and separated by an interface. By carefully choosing the matrix, the reinforcement, and the manufacturing process that brings them together, the engineers can tailor the properties to meet specific requirements [23]. The modifications inside the new nanocomposite are explained by an increase in the surface/volume ratio, which increases the contact area between the reinforcement and the matrix.

#### 2.1.1. Polymer Matrix

Any material can serve as a matrix material for composite; however, the most common materials employed as a matrix are ceramics, metals, and polymers. Most matrix materials on the composites market are polymers [24]. Polymer matrices (PMs) have been widely used as a matrix for composites production because they have great properties, including excellent toughness and adhesion [25]. The polymer matrix is the continuous phase in the composites used to hold the reinforcing agent in its place. Additionally, its properties determine most of the degradative processes (delamination, impact damage, chemical resistance, water absorption, and high-temperature creep) [26]. 

##### Hydrogels

One of the most common hydrophilic PMs is those known as hydrogels. A hydrogel is a tridimensional polymeric structure with swelling and collapse properties, flexibility, biodegradability, biocompatibility, and softness [27,28,29]. Some hydrogels can respond to external stimuli such as pH, temperature, electricity, light, magnetism, and biological molecules such as enzymes during the swelling and shrinking [30]. In electronics, conductive hydrogels are good prospects for use in this field due to their remarkable properties, such as good electronic properties, mechanical flexibility, ease of processing, and the previously mentioned biological characteristics, which can be used to satisfy specific requirements and demands [31,32]. The main disadvantage of these polymeric materials is their relatively low (compared to metals) temperature resistance limited by the matrix, which can be enhanced with reinforcement [20].

##### Conducting and Semiconducting Polymers

Scientific and technological development has given rise to two types of electronic conductive materials with a polymeric matrix: extrinsic and intrinsic conductors. Extrinsic conductors have a polymer matrix with composite materials made up of a polymer, generally thermoplastic, and a filler. The extrinsically conducting polymers are insulators that enable their electrical conduction by introducing a conducting mediator inside their polymeric chains [33].

**Figure 1:**
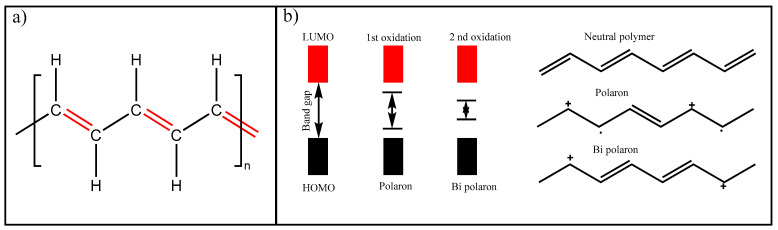
(**a**) Graphical representation of intrinsically conducting polymers and (**b**) p-doping process.

Intrinsic conducting polymers are also called conjugated polymers since a generalized characteristic is the successive alternation of a single bond with double bonds (Figure 1a) along the main chains. In that context, the inherent electrical conductivity can be explained by the facility of π electron to be transferred to vicinal carbon allowing the electron flow [33,34]. However, this mechanism produces a low conductivity (between 10^−10^ to 10^1^ S/cm): thus, it is required to generate a charge carrier by oxidation (p-doping) or reducing (n-doping) the polymer using chemical or electrochemical methods, which in most of the cases are reversible processes [35,36]. Doping improves the conducting character of polymers considerably in that way is possible to generate a semiconductor with relatively high conductivity (1 to 10^4^ S/cm) because this modification produces a substantial change in the band-gap value [37]. The band-gap theory approach will be used to understand the doping mechanism. As Figure 1b shows, an electron is extracted from the HOMO (p-type doping) of the valence band in the oxidation. Additionally, a polaron (cation-radical specie) is created when two electronic levels inside the band gap are generated, reducing its value. A bipolaron (bi-cationic specie) is formed in second oxidation, and the band gap is reduced even more [38]. These mentioned charge carriers allow a strong delocalization of electrons that can now flow freely along with polymer conjugation without much resistance to the band gap. In some cases, the conductivity observed in intrinsically CP is close to metallic materials [39]. Regarding reduction (p-type doping), it is not ccommon because the intrinsically conducting polymers are electron-rich components, which means that they will not allow accepting a negative charge.

**Figure 2:**
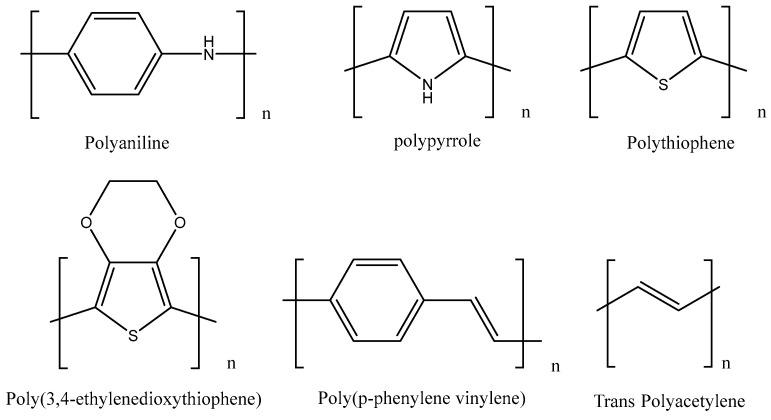
Structure of the most used intrinsically conductive polymers.

In this sense, semiconducting polymers, that is, polymers with a broadband gap and lesser degree of conjugation, have acquired great relevance in electronics due to their rich optical and electrical properties, they are used as organic photovoltaics [40], organic light-emitting diodes, stretchable electronic devices [41], supercapacitors [42], fuel cells [43], and electrochemical sensors [18]. Figure 2 shows some examples of intrinsically conducting materials that will be observed as a part of nanocomposites in the future in this document.

#### 2.1.2. Reinforcement

The reinforcement material is added to the matrix material to enhance the physical properties of the final composite material [44]. In other words, the reinforcing phase provides strength and stiffness because the reinforcement materials are more rigid, stronger, and stiffer than the matrix [45]. These fillers or reinforcement materials might be classified according to their chemical nature and physical structure, but usually, they are classified based on the shape of the particles [46]. Reinforcement is typically added to polymeric matrices to improve their chemical and physical properties. Of these properties, the optimization of the mechanical characteristics has been the most desired objective, leading to the development of reinforcements that range from inorganic fibers (glass and carbon fibers) to organic (aramid) and inorganic particles (SiO_2_, Al_2_O_3_, CaCO_3_), carbon black, and silicate sheets [47]. Despite having materials with exceptional properties, the practical applications are reduced by factors that greatly increase their costs, such as the difficulty of manufacture or the incompatibility between materials.

##### Nanoparticles as Reinforcement

Nanoparticles (NPs) are ultrafine particles that have sizes between 1 and 100 nm [48]. These NPs can serve as a filler in the polymeric matrix. The NPs exhibit higher specific surface area, surface energy, and density than microparticles; hence lower nanofiller concentrations are needed to attain properties comparable to or even better than those obtained by conventional micro filler loadings, which facilitate processing and minimize the increase in composite weight [49]. After adding NPs to the PM, a nanocomposite is obtained, which will present a specific structure with physical properties that depend on the content and type of nanometric charge incorporated [50]. NPs display many atoms that make them up and belong to their surface. Therefore, these atoms can easily interact with the matrix where they are embedded [51]. A significant reason for using NPs is to reduce the cost of the composite. They are also readily available in the required quantities, sizes, and shapes [52].

### 2.2. Magnetic Nanoparticles 

Magnetic nanoparticles (MNPs) show excellent features such as small size, high surface area, an active surface that can successfully be modified, low toxicity, and superparamagnetism (magnetic behavior with characteristics of ferromagnetism and paramagnetism) [53]. Currently, magnetic nanostructures exhibit a great variety of applications ranging from electronics, mechanics, and optics to reaching more complex areas such as biomedicine, where superparamagnetic nanoparticles are used for the diagnosis [54] and treatment of diseases by the transport and release of drugs [55] and immobilization and magnetic separation of biological entities or treatment of tumors using hyperthermia therapies [56]. However, the functionalization of the MNPs with organic and inorganic materials is indispensable for their applications [57].

The size reduction brings about a change in the magnetic behavior of the material since it goes from being ferro- or ferrimagnetic to superparamagnetic, where, due to the effect of thermal energy, the magnetic moment of each particle fluctuates in the direction being the net magnetic moment equal to zero [58]. The unique physicochemical properties of MNPs, especially their large surface areas, ease of synthesis and modification, and inherent superparamagnetic properties, could lead to improved technologies. Moreover, these MNPs present an excellent capability to achieve a synergic union with other compounds, such as polymers [57].

#### 2.2.1. Types of Magnetic Semiconductor Nanoparticles

##### Metal and Metal Oxide Nanoparticles

**Figure 3:**
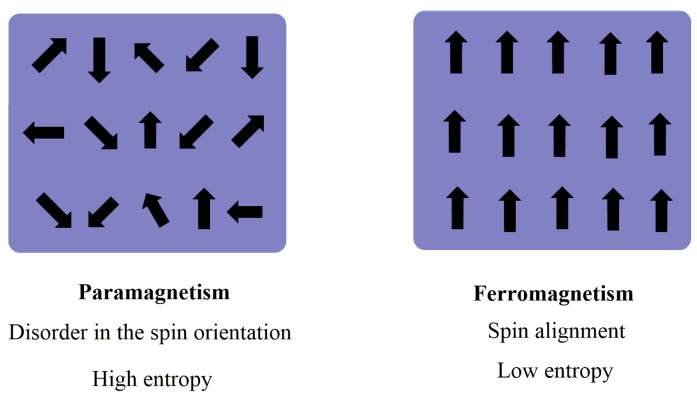
Graphic illustration of paramagnetic and ferromagnetic behaviors.

Metal oxides have always aroused technological and scientific interest due to their various properties (optical, magnetic, electrical, and some others) and their general characteristics of hardness, thermal stability, and chemical resistance [17]. Metallic elements can form many oxides capable of adopting many structural geometries and with electronic properties that can exhibit conductive, semiconductor, and even insulating characteristics. Ferromagnetic oxides are the preferred materials for information storage and transmission. Transition metal oxides such as Fe, Ni, and Co have a peculiar magnetic behavior since they are ferromagnetic at low and ambient temperatures and show paramagnetism at high temperatures, as is shown in Figure 3 [59,60]. Some oxides with variable electron mobility are used as semiconductors (V_2_O_5_) or superconductors (YBa_2_Cu_3_O_7_). Ferroelectric and dielectric oxides with perovskite structure (BaTiO_3_, PbZrTiO_3_) are extensively used in electronic devices [61,62,63,64].

At the macroscopic level, metal oxides constitute robust and stable systems with well-defined crystallographic structures. In nanomaterials, however, changes in thermodynamic stability can be observed, including modifications in cell parameters and in the crystal structure of these materials. As a result, phases with very low macroscopic stability can be formed into highly stable nanostructures (TiO_2_, VO_x_, Al_2_O_3_, MoO_x_) [65]. Non-stoichiometric structure phenomena (widespread in manganese oxides) and the formation of non-existent phases at the macroscopic level can also be observed.

Metal oxides at the nanoscale exhibit unique chemical and physical properties due to their limited size and the high density of active sites on their surface. The surface atoms, with a coordination number lower than those located inside the mass, together with the existence of oxygen vacancies on the surface of the oxide, give rise to structural arrangements that generally improve the chemical activity of the systems involved. The decrease in size strongly influences the conductivity and chemical reactivity properties [66]. The strongly modified surface properties give rise to solids with unprecedented acid/base and adsorption characteristics. Furthermore, the oxides of the metals, as mentioned earlier, are used in biomedical and industrial applications due to their magnetic and electronic versatility [66,67,68].

##### Ferrite

Ferromagnetic oxides, commonly called ferrites, are ceramic materials composed mainly of iron oxide combined with other metallic elements. They are ferromagnetic materials; they can be magnetized or attracted by magnets [69].

The magnetic properties of ferrites derive from their crystalline structure, where the metallic atoms occupy well-defined positions about the oxygen atoms. Soft ferrites generally have a cubic structure of the generic spinel-type MFe_2_O_4_, where M represents one or more of the transition metals Mn, Fe, Co, Ni, Cu, or Zn. Several scientists and researchers have reported some properties and applications of these particles with ferromagnetic properties [70,71].

##### Manganite

Manganites are compounds of the type A_1-x_A_’x_MnO_3_, where A is rare-earth (La, Pr, Y) and A’ is alkaline earth (Sr, Ca, Ba). Type A cations have a valence of +3, and A’s have a valence of +2 [72]. Therefore, when substituting a fraction x of cations, A for A’ in the AMnO_3_ material, the valence of an equivalent fraction of Mn ions changes from +3 to +4. The crystalline structure is perovskite-type with distortions concerning the cube according to the A and A’ ions present, giving rise to orthorhombic or rhombohedral structures [73]. A great variety of magnetic behaviors have been found in different manganites and, associated with them, different properties of electrical transport and presenting various fields of industrial and magnetic applications [74,75].

**Figure 4:**
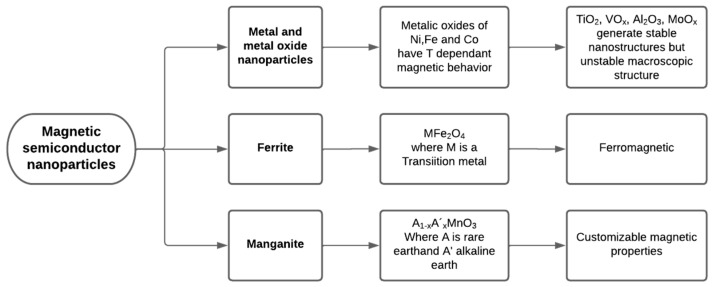
Summary of magnetic semiconductor nanoparticles.

Magnetic semiconductor nanoparticles discussed in this section with characteristics and applications are summarized in Figure 4.

### 2.3. Polymer Nanocomposites

**Figure 5:**
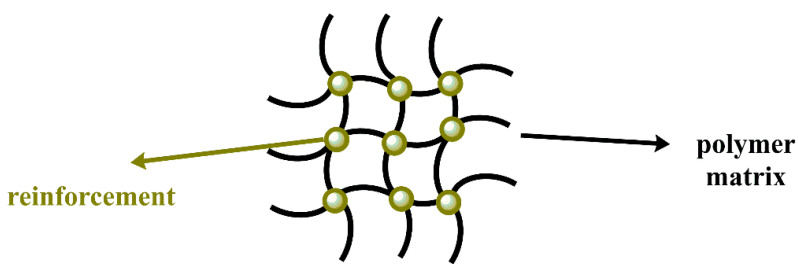
Polymer nanocomposite.

Currently, researchers have led studies approaching the use of conventional polymers as one of the components of NCs, forming in a particular type of hybrid materials called polymer nanocomposites (PNCs) (Figure 5) [76]. Moreover, the incorporation of NPs with remarkable properties in polymeric matrices has gained great interest thanks to the significant changes in thermal, optical, electrical, and magnetic properties that are conferred on the final nanocomposites compared to the pure matrix [61]. The interesting properties of the PNCs will depend either on the morphology, surface properties, and arrangement, in a wide range of the NPs, as well as on the structure of the NCs, which depends on the compatibility between the organic and inorganic phase and the mixing, and the dispersion method used during its preparation. One of the significant challenges when preparing PNCs is the homogeneous dispersion of nanofillers in a polymer [77].

#### Magnetic Polymer Nanocomposites

Polymers can serve as an excellent matrix for the processing of NCs; however, they have no magnetic properties. Therefore, incorporating nanofillers with magnetic properties is an excellent solution to improve the characteristics of the NCs. Nanosized particles of ferromagnetic and ferrimagnetic materials have been incorporated into extended matrix materials to create integrated functional systems with additional magnetic properties [78].

Recently, polymeric nanocomposites with magnetic properties have aroused great interest in the scientific value of understanding their properties and their numerous applications [79]. Among them, the most common are in the fields of sensors and transducers, electronic devices, magnetic storage, electromagnetic and microwave absorption, and magnetic actuators [80,81]. In the first decade of the 2000s, studies were carried out on obtaining these materials by incorporating iron carbide particles in micrometric sizes into elastomeric matrices, where the increase in the elastic modulus of the magnetic elastomer was evidenced under the application of an external magnetic field [82]. Furthermore, Muñoz-Bonilla et al. reported using magnetite NPs to obtain NCs using polyaniline as a conductive polymer matrix with superparamagnetic behavior for electromagnetic shielding applications [83].

As mentioned earlier, ferrite combines magnetic material properties with those of an electrical insulator. It is a good option due to its flexibility to modulate their magnetic and electromagnetic properties, low cost, and ease of obtaining [84]. Ferrite-reinforced composite materials vary in their properties depending on the amount of ferrite added to the polymer matrix. Bellucci et al., for example, produced natural rubber nanocomposites reinforced with zinc ferrite nanoparticles using a rubber mill [85]. The study shows that the magnetic hysteresis loops are tight, which shows little material loss during the magnetization/demagnetization process. The remanence magnetization values are small, as are those of the coercive field, a typical behavior of soft ferrites.

Hard ferrites such as barium ferrite (BaFe_12_O_19_), unlike soft ferrites, have broad hysteresis loops. These materials have been used to make compounds with matrices of natural rubber or reused rubber from used tires [86,87]. In these works, the saturation magnetization and remanence magnetization vary linearly with the content of the reinforcement, as do materials reinforced with soft ferrites. The main difference lies in the wide hysteresis loops typical of hard ferrites. Makled et al. found that the coercive field tends to decrease for charges equal to or greater than 100 phr due to the increasing magnetic interaction between the ferrite particles as the average distances between them decrease [86].

Conductive reinforcements such as carbon-based composites are required to have adequate reflection values of the incident electromagnetic radiation in a polymeric matrix composite material. The composite material must have a reinforcing material with abundant electric or magnetic dipoles to obtain significant absorption values of the same radiation. Sunny et al. synthesized nanocomposites from natural rubber and nickel ferrite (NiFe_2_O_4_) to evaluate their magnetic and electromagnetic properties, finding that there is a constant increase in saturation magnetization and dielectric permittivity as the content of the reinforcement increases, as with magnetic permeability, although the latter decreases with increasing frequency [88]. For its part, the highest reflective losses that quantify the electromagnetic energy absorbed or dissipated by the composite material in the event of incident waves, for this case, were −5.9 dB for low frequencies (2–3.5 GHz) and −16 dB for high frequencies (9.66 GHz), both for a 120 phr nickel ferrite load and 12 mm thick sheets.

Magnetite is a soft ferrite commonly found in nature and easily obtained in the laboratory; it has ferrimagnetic properties and acceptable electrical conductivity values generated by its inverse spinel structure, where the divalent cation Fe^2+^ is found in the octahedral sites, while the trivalent cation Fe^3+^ is found in the tetrahedral and octahedral sites. The constant jump of electrons between the Fe^2+^ and Fe^3+^ ions found at the octahedral sites impart some metallic properties to the magnetite even at room temperature. Kong et al. manufactured and evaluated the microwave absorption properties of compounds made from natural thermoplastic rubber reinforced with magnetite nanoparticles (Fe_3_O_4_) in different concentrations of 4–12 wt% using an internal mixer [89]. The thermoplastic rubber matrix was synthesized from polypropylene (PP) and natural rubber. The real and complex permittivity values, the imaginary permeability, and the saturation magnetization values increase while the real permeability decreases with increasing reinforcement content. The microwave absorption properties and the absorption bandwidth of the compounds can be easily modulated by changing the magnetite concentration in the matrix and the thickness of the sample.

Ramapo et al. also found that high dielectric losses and high permittivity values are obtained at low frequencies when high concentrations of magnetite are added to thermoset matrices (40 vol%) [90]. The magnetite particles were processed through a ball mill to obtain micrometric sizes. Later these particles are added to epoxy resin matrices. The method of obtaining the particles generates a wide distribution of sizes and appearance of the particles that directly affect the shielding properties of the material.

Other properties such as the volumetric resistivity of this type of material depend on the magnetite content added to the matrix. Weidenfeller et al. added different proportions by volume of magnetite in polypropylene and polyamide matrices using an extruder as a manufacturing tool [91]. A decrease of up to seven orders of magnitude was evidenced for magnetite contents of 47 vol%. For volume contents above 30%, contact points begin to exist inside the matrices, which translates into a noticeable drop in the resistivity of the compounds [91]. The different grain sizes and the additives used during mixing do not influence the conductive behavior of the compound. Similar results of volumetric resistivity were obtained in another study in which compounds with high-density polyethylene matrices were reinforced with magnetite. They found a significant drop in the material resistivity of up to six orders of magnitude for samples of pure HDPE, up to magnitudes of 106 Ω*cm for samples with a content of 40% by volume of magnetite. In this same work, it was also observed how the saturation and remanence magnetization of the compound increased linearly with the amount of reinforcement added to the matrix [92].

## 3. Synthesis of MNCs

The synthesis of hybrid nanocomposites (magnetic and polymers) has significant advances in the last years. New strategies have been developed for magnetic nanocomposites with desirable properties. However, the homogeneity of MNPs into the polymeric matrix is still a challenge. Several processes have been designed to accomplish this objective, such as molding, coprecipitation, in situ precipitation, chemical vapor deposition, blending, and grafting onto the method. Practically, magnetic composites can be synthesized by embedding magnetic particles into a non-magnetic matrix [93].

### 3.1. Molding

**Figure 6:**
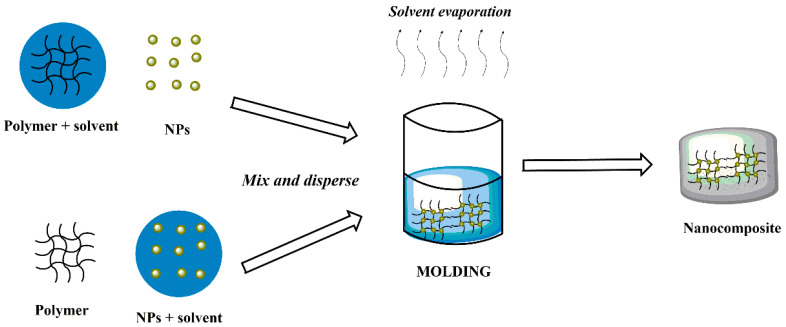
Schematic representation of molding technique.

Molding is a widely used fabrication process of magnetic composites that is accomplished by mixing magnetic fillers and polymeric precursors thoroughly and curing them to form specific shapes or structures in molds [94] (Figure 6). There are several types of molding processes, such as injection [95], resin transfer [96], and compression molding techniques [97], which practically use a mold that is filled under pressure and temperatures. Chen et al. reported the synthesis of highly crosslinked poly(cyclotriphosphazene-co-4,4′-sulfonyldiphenol) (PZS) used to coat ferric oxide (Fe_3_O_4_) nanoparticles directly and then were loaded with carbon nanotubes (CNTs) to get CNTs/Fe_3_O_4_@PZS as the photothermal magnetic filler. The PDMS/CNTs/Fe_3_O_4_@PZS surfaces with micron-scale truncated cones were prepared using compression molding and magnetic attraction [98].

### 3.2. Coprecipitation

**Figure 7:**
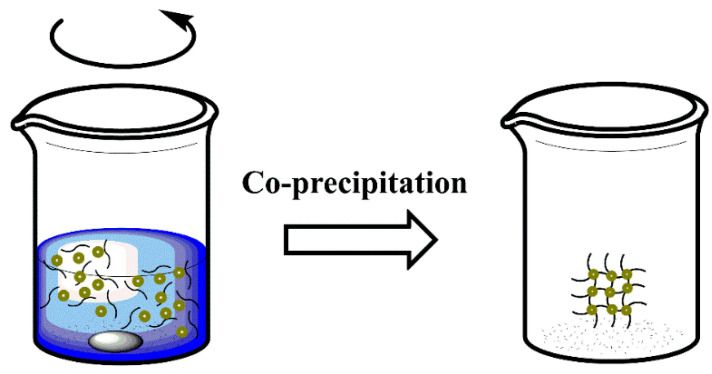
Schematic representation of coprecipitation method.

Coprecipitation is a facile and convenient approach to preparing MPNs [99] (either Fe_3_O_4_ or γ-Fe_2_O_3_) from aqueous Fe^2+^/Fe^3+^ salt solutions [100]. This process generally involves forming simultaneous nucleation, growth, coarsening, and agglomeration processes [79] (Figure 7). Generally, MNPs synthesized by coprecipitation will result in polydispersed particles that are spherical and have a size distribution between 5 and 40 nm [57,59]. Moreover, magnetic nanocomposites have been studied by the coprecipitation method, which is generally synthesized from salt species like Fe^2+^ and Fe^3+^ in alkali solution and under non-oxidizing conditions [101]. For example, Mehra et al. used an in situ coprecipitation method to achieve a homogeneous melamine-cyanurate (MC) distribution in a polymer matrix. As a result of this incorporation, a 65% enhancement of thermal conductivity was achieved (0.66 W/m·K), offering a new strategy for developing new thermally conductive materials [102]. 

### 3.3. In Situ Polymerization

**Figure 8:**
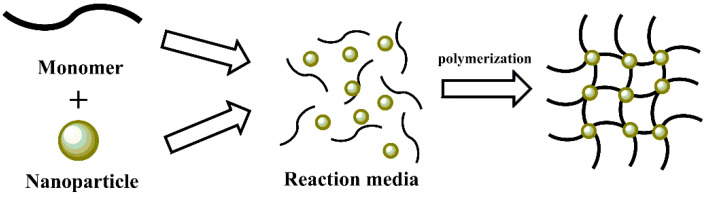
Schematic representation of in situ polymerization.

The in situ polymerization method is widely used and reported in the literature to synthesize MNPs based on their application in many fields [103,104,105]. This simple and straightforward method loads MNPs into a polymeric matrix because this method involves the inclusion of nanoparticles into a polymer matrix in the presence of a precipitation medium [106] (Figure 8). The selective precipitation of small amounts of inorganic nanoparticles within the porous matrix reduces the accessible pore volume [107]. Pu et al. synthesized a superparamagnetic graphene oxide (GO)/Fe_3_O_4_ nanocomposite by a facile in situ coprecipitation strategy for bioremediation [108]. Wang et al. reported the synthesis of novel composite hydrogels using in situ method. First, they proposed the synthesis of polypyrrole (PPy) and Fe_3_O_4_ nanoparticles and then the inclusion of this composite within the polyvinyl alcohol (PVA) matrix. The result was a hybrid hydrogel, named Fe_3_O_4_/PPy/PVA hydrogel, which shows interesting mechanical, conductive, and magnetic properties, making it a potential candidate for biomedical electronic devices [109]. Additionally, Hermán et al. prepared a core double-shell cobalt/graphene//polystyrene nanocomposite (Co/C//PS) by in situ polymerization, obtaining highly protected Co particles in the nanocomposite with a high mass-magnetization, that is, ~49 emu/g for 94% wt Co/C [110]. In another interesting research study, Shabzendedar et al. synthesized a superparamagnetic core-shell nanocomposite of poly(*m*-aminobenzenesulfonic acid) (PABS) embedded with Fe_3_O_4_ nanoparticles by an in situ polymerization method. This novel material exhibit excellent magnetic properties showing a saturation magnetization value of 40 emu/g and improved power-conversion efficiency (η = 4.24% and 660% enhanced efficiency), which can be exploited in polymer solar cells [111]. 

### 3.4. Chemical Vapor Deposition (CVD)

**Figure 9:**
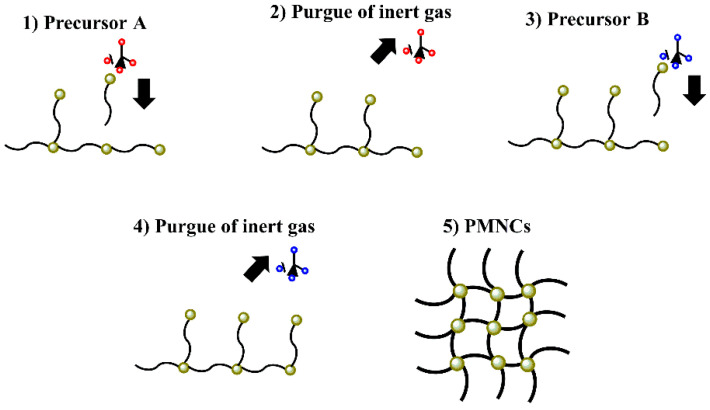
Schematic representation of the CVD method.

CVD is utilized to produce composite material films and infiltrate fabric to produce different nanomaterials [112]. This technique involves the deposition of a solid in a matrix through a chemical reaction considering one or several gases (Figure 9). There are few reports on magnetic oxide films fabricated using CVD compared to other methods; however, some interesting magnetic materials can only be deposited via CVD [113]. In this technique, the precursors, gas or vapor, can react or decompose on the preselected substrate at high temperatures and vacuum in a chamber [114]. Several gases are admitted into the vacuum chamber through the inlet, and after dissociation between the species, the newly formed chemical molecules are deposited on the heated substrate [115]. CVD has proved to be a method that can produce iron oxide nanocomposites with high performance as a solid material and produce one-dimensional nanomaterials with a high purity [101].

### 3.5. Spin Coating

**Figure 10:**
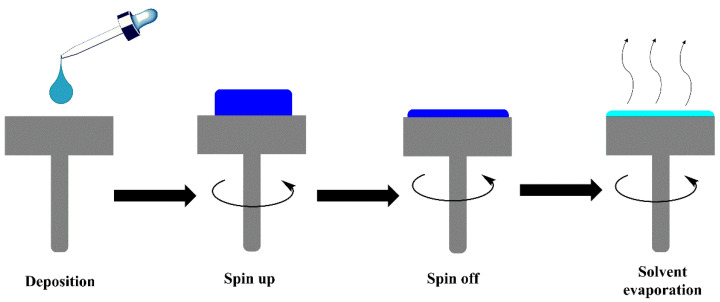
Schematic representation of Spin coating method.

Spin coating is a technique used in the microelectronics industry to disperse thin films or nanoparticles on solid substrates through centrifugal force. The rotation continues until the applied solvent evaporates and the thickness of the layer on the nanocomposite is reached [116,117,118] (Figure 10). Using this method, Sasikumar et al. prepared a highly sensitive resistive type of humidity sensor of TiO_2_/PANI nanocomposite. The exciting properties obtained can be reflected in the obtained values of room temperature resistivity (2.56 × 10^3^ Ω.cm), fast response (20 s), and recovery time (15 s) [119]. In another research, Chou et al. dispersed Fe_3_O_4_ magnetic nanoparticles in poly(3-hexylthiophene-2,5-diyl) (P3HT), obtaining a novel polymer ferromagnetic semiconductor with a coercivity of 300 Oe. This value indicates a strong interaction between matrix and reinforcement, which was achieved using this method [120]. Additionally, Sharif et al. incorporated Mg_0.5_Zn_0.5_Fe_2_O_4_ nanoparticles by spin coating method into a PVA network matrix, obtaining good dielectric properties with potential applications in memristors and random access memories (RAM) [121]. 

## 4. Electronic Applications

In recent years, nanotechnology has been widely linked to the design and production of advanced devices focused on electronic applications. In this sense, magnetic polymer nanocomposites have been very useful due to their versatility, low weight, low cost, and easy preparation [122,123,124]. This section details certain applications that have found these promising materials.

### 4.1. Supercapacitors

Supercapacitors are electrochemical capacitors with energy densities an order of magnitude greater than the densities of conventional capacitors. Compared to batteries, these devices have low internal resistance, which means they can achieve high power densities [122]. Currently, supercapacitors are used to stabilize varying charges and to provide fast charging for mobile electronics. Additionally, these capacitive devices serve as a power buffer to mitigate voltage swings. Supercapacitors will not replace batteries in most applications, but they are likely to co-exist with them [125,126]. According to Altai et al., supercapacitor devices will be used where high levels of instantaneous power are required and fast recharging. On the other hand, batteries will be needed when renewable energy sources are intermittent [127].

To meet these needs, metal oxide coupled conducting polymer nanocomposites are a pseudocapacitive material that exhibits high-performance electrochemical properties for charge storage applications [128]. Conductive polymers such as polyaniline, polypyrrole, polythiophene, and some others, have been shown to have great flexibility, controllable thickness, and a high conductivity value (>10^3^ S/cm) but lower stability [129]. In this sense, Prasankumar et al. prepared a polyaniline (PANI)/Fe_3_O_4_ nanocomposite by an in situ method. This nanocomposite showed improved electrochemical properties and high specific capacitance value, that is, 572 F/g at 0.5 A/g, and pronounced cycling stability (>5000 cycles at 1 A/g) with excellent capacitance retention of 82%, proving that this material can be exploited as a highly efficient electrode for supercapacitor [130]. Additionally, Sadeghinia et al. deposited BaFe_12_O_19_ nanoparticles into the PANI matrix to obtain a nanocomposite with a specific capacitance in the range of 225 and 330F/g, depending on the content of the reinforcement [131]. On the other hand, Asen et al. incorporated Cr_2_O_3_-graphene oxide into conducting polymer matrices such as polyaniline (PANI) and polypyrrole (PPy). The materials obtained showed high specific capacitance values of 525 F/g for the PANI-based nanocomposite and 495 F/g for the PPy-based nanocomposite, both at 5 A/g. Interestingly, these nanocomposites retain up to 84 and 80% of the initial capacitance after 4000 charge–discharge periods, suggesting improved electrochemical stability [132]. 

Among metal oxides, titanium oxide (SnO_2_) is an *n*-type semiconductor with a band gap of 3.6 eV, and its inclusion in PANI arrays has demonstrated applications in electronics [133]. Utilizing cyclic voltammetry, Prasanna et al. found that this inclusion to form a nanocomposite shows a specific capacitance of 337 F/g at a current density of 0.2 A/g and capacitance retention of 73% after 2000 cycles [134]. Even the inclusion of this reinforcement within a ternary nanocomposite shows improved electrochemical properties, as demonstrated by Ghebache et al. in the preparation of a nanohybrid electrode consisting of HY zeolite/SnO_2_/PANI, which shows a maximum capacitance of 1085 F/g at 5 mV/s, a good result derived from the synergistic effect with the complementary properties of both components of the filler [135]. Additionally, Kandasamy et al. synthesized a ternary nanocomposite consisting of carbon nanotubes/SiO_2_/PANI. This nanocomposite showed a specific capacitance value of 221 F/g at a current density of 2 A/g, with 95% capacitance retention after 1000 charge–discharge cycles due to an increase in quantum capacitance induced by interactions between PANI and the dual reinforcement [136].

Another conductive polymer matrix widely used for the development of supercapacitors is poly(3,4-ethylene dioxythiophene) (PEDOT), a polymer that has some advantages over other conductive polymers due to its long-term electrical stability, low band gap, improved mechanical properties, lower cost, and good electrochemical activity [137,138]. In this way, Ates et al. synthesized a new nanocomposite electrode by incorporating reduced graphene oxide (rGO) and tin oxide (TiO_2_) into the PEDOT matrix. This inclusion increases the specific capacitance to 652 F/g at 1 mV/s in a ratio of [rGO]/[TiO_2_] = 1/5. Another result for SC varied depending on the content of filler, that is, [rGO]/[TiO_2_] = 1/2 (SC = 475.33 F/g), [rGO]/[TiO_2_] = 1/1 (SC = 48.02 F/g), rGO/PEDOT (SC = 114.09 F/g), which demonstrates the distinct possibilities of this new nanocomposite to be incorporated as future supercapacitor application [139]. Gupta et al. replaced TiO_2_ with Mn ferrite (MnFe_2_O_4_) by an in situ polymerization, obtaining a material with EDLC and pseudocapacitive behavior with a good specific capacitance of 298.97 F/g at 1 A/g, and retention of these values at 81% over 5000 cycles. This ternary nanocomposite is a promising possibility as an electrode for supercapacitor fabrication [140]. 

### 4.2. Sensors

Industrial sensors are a crucial part of factory automation and Industry 4.0. A sensor is an electronic device that detects and responds to input from the physical environment and converts these output signals into a human-readable display. In industrial automation, sensors play a vital role in making products exceptionally automatic. They can also confer improved performance for energy sources such as fuel cells and batteries, and solar power, thus conferring better lifestyles for human beings [141,142,143].

With the growing industrial population, several environmental challenges have arisen due to the emission of toxic and dangerous gases [144]. For this reason, its detection by employing sensors has become essential. With this approach, Saaedi et al. dispersed zinc oxide (ZnO) into the PANI matrix, obtaining a nanocomposite to detect methanol, a highly volatile, flammable, and toxic alcohol. This study showed that the nanocomposite synthesized at a magnetic flux of 0.5 T obtained a resistance of 865 k, response time of 18.2 s, and recovery time of 5.1 s [145]. Wang et al. developed a material with potential application in detecting ammonia, a harmful gas to human health. For this purpose, Wang et al. synthesized a CuFe_2_O_4_/PANI nanocomposite, which showed an improved response time (67%) compared to pristine PANI and CuFe_2_O_4_ films [146]. Additionally, Husain et al. prepared a polythiophene/zirconium oxide (PTH/ZrO_2_) nanocomposite with high thermal stability and high electrical conductivity (9.42 × 10^−4^ S/cm) that can be applied to the detection of ethane [147].

The detection of contaminants such as insecticides is important for their subsequent removal. In this sense, this review shows two striking studies in which sensors based on magnetic polymer nanocomposites are used. The first was developed by Miao et al., who synthesized a nanocomposite based on Fe_3_O_4_ and polydopamine to detect dichlorodiphenyltrichloroethane (DDT) insecticide. This obtained sensor exhibited a limit of detection of DDT of 6 × 10^−12^ M, which was proved to be more efficient than other sensors [148]. Sohrabi et al. prepared an imprinted polymer nanocomposite for adsorption of diazinon insecticides. It was achieved by including graphene oxide, clay, and Fe_3_O_4_ into polydiacetylene (PDA), showing a magnetic saturation of 8.28 emu/g, detection of diazinon at 1.45 ppm, and adsorption capacity of more than 98% up to nine cycles of adsorption/desorption process [149].

### 4.3. Light-Emitting Diodes (LED)

A light-emitting diode (LED) is a light source that emits photons when an electrical current of very low intensity is received. An LED is usually encased in a colored plastic material that accentuates the length of light generated by the diode and helps focus the light into a beam [150]. Observing these properties, LEDs became popular, and advantages were found over other technologies. These advantages include long life, low power consumption, high level of efficiency, small dimensions, immediate light when turned on, and resistance to shocks or vibrations, which can be used in smart TV screens, smartphones, PC monitors, billboards, and some others [150,151].

This technology has been studied over the years. It has been tried to couple the use of magnetic polymer nanocomposites-based LED, due to its versatility and properties, including magnetic and optoelectronic properties [152]. In this sense, Skoda et al. synthesized a nanocomposite with deposition of Co_x_Zn_1-x_O (x = 0.01, 0.05, and 0.1) nanoparticles into poly[2 -methoxy-5-(2-ethylhexyloxy)-1,4-phenylenevinylene] (MEH-PPV). The inclusion of Co^2+^ into the ZnO lattice provides different optoelectronic properties such as band gap narrowing and luminescence quenching. Then, the addition of obtained nanoparticles into the polymer matrix gives a homogeneous solution for spin coating of thin nanocomposite layers, with application in optoelectronic devices, including hybrid polymer light-emitting diodes [153]. In the same research team, Jamatia et al. included Fe_x_Zn_1-x_O in the stoichiometric relationships in the previous research. By a spin coating method, nanoparticles were dispersed into MEH-PPV, showing a significant enhancement of electroluminescence of the PLED devices due to the inclusion of nanoparticles into the active layer [154].

Including some relevant reinforcements into polymer matrices has been widely reported to obtain improved optoelectronic properties. Kumar et al. synthesized polymeric nanocomposites based on poly(*p*-phenylenediamine) filled with ZnO, Fe_3_O_4,_ and TiO_2_ nanoparticles, describing an electrical conductivity of 10^−7^ S/cm, which makes it a semiconducting material. With complementary studies, the Fe_3_O_4_ nanocomposite exhibits improved soft magnetic properties reflected from the coercivity, retentivity, magnetization, and moment [155]. In another research team, Hadavand et al. deposited CdS/ZnS nanoparticles into poly(3,4-ethylene dioxythiophene) polystyrene sulfonate (PEDOT: PSS) polymer as a hole transport layer in samples of LEDs. The measurements of voltage-current density show that the turn-on voltage of obtained LED (0.4% of nanocomposite included) was decreased from more than 3 V for pristine LED to 1 V [156]. Additionally, Chen et al. fabricated a nanocomposite encapsulating CsPbX_3_ (X = Cl/Br, Br, Br/I) perovskite nanocrystals into superhydrophobic matrix-like poly(styrene-ethylene-butylene-styrene) (SEBS). The obtained CsPbX_3_@SEBS flexible films were used as color conversion materials to fabricate LED devices, exhibiting a wide color gamut (113% according to the National Television System Committee), a promising value for highly efficient color conversion materials for white LED [157].

On the other hand, the inclusion of other particles, like quantum dots (QDs), into polymer matrices can be beneficial to obtaining better light performance due to their great potential in optoelectronic devices, derived from their excellent optical performance [158]. Zhu et al. synthesized a nanocomposite from perovskite (FAPbBr_3_) QDs, as filler, and poly(methylmethacrylate) (PMMA), as a matrix. The final product showed a Lumen Efficiency of 80.4 m/W and a Color Rendering Index of 90, which satisfy commercial expectations and suggest application as light-emitting diode material [159].

### 4.4. Solar Cells

**Figure 11:**
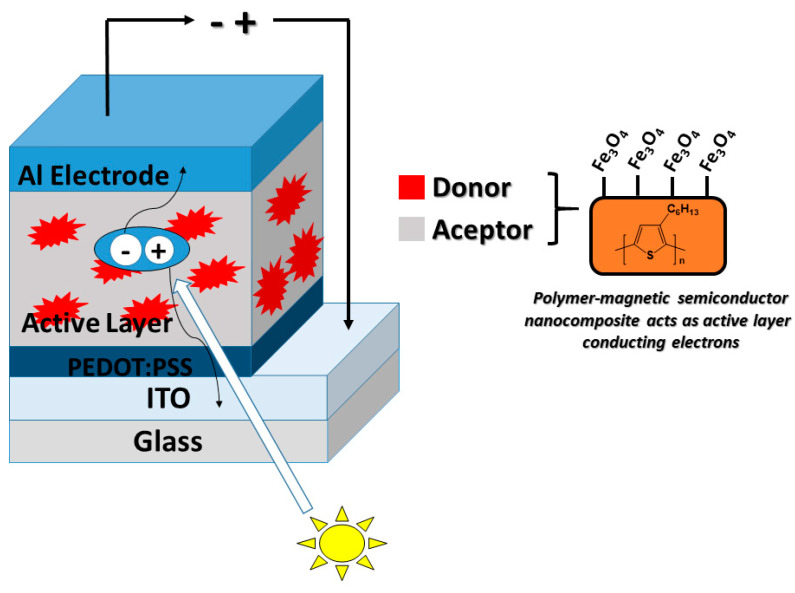
The basic scheme of an organic solar cell.

Solar cells are energy converters in the form of electromagnetic radiation into electrical energy. Once this radiation makes contact with the semiconductor material (donor in the active layer), it transforms it into electrical energy in the form of direct current to be used immediately and, in turn, can be stored in a battery bank through load control [160] (Figure 11). Within these materials for harnessing solar energy, those solar cells based on organic semiconductors represent a promising technology due to their low cost, low weight, and versatility. Thus, polymer solar cell (PSC) devices have gained ground by using a π-conjugated polymer donor with a nanoscale coating; it forms a nanocomposite [161]. These materials have advantages compared to conventional solar cells, such as improved stability and efficiency [160,162]. Meng et al. describe the fabrication of composite nanofibers based on Fe_3_O_4_ as filler and blends of regio-regular poly(3-hexylthiophene) (P3HT) as the matrix. The resulting hybrid nanocomposite shows electronic properties related to the matrix and improved magnetic responsiveness. Despite having controlled and stable morphologies, there is no significant improvement in organic solar cells’ performance with this obtained material [163].

Polymer matrices, magnetic fillers, properties of nanocomposites, applications, and corresponding references explained in this section are summarized in Table 1.

## 5. Perspectives and Conclusions

The incorporation of nanoparticles with remarkable properties in polymeric matrices has gained great interest thanks to the significant changes in thermal, optical, electrical, and magnetic properties that are conferred on the final nanocomposites compared to the pure matrix. These unique properties depend not only on the morphology (size and shape), surface properties, and organization in a wide range of the nanoparticles but also on the structure of the nanocomposites. Nanocomposite materials with inorganic–organic phases can no longer be solely considered reinforced material. It will depend on the compatibility between the organic and inorganic phases and the mixing and dispersion method used for its preparation.

The brittleness, great weight, and processing difficulty of conventional solid materials with magnetic and conductive properties limit their use in many potential applications. On the other hand, polymeric materials are lightweight, easily processable, and mechanically robust, but generally not magnetic. Therefore, the combination of polymers and inorganic nanofillers with magnetic properties is presented as a solution for integrating magnetic properties into a soft matrix.

It is essential to provide nanocomposites with good processability properties to exploit the great potential of their technological applications. This factor, in recent years, has led researchers to use conventional polymers as one of the components of nanocomposites, resulting in a particular type of hybrid material. Polymeric nanocomposites with magnetic properties have attracted great interest, not only for the scientific value of understanding their properties but also for their many applications. The most common are in the fields of sensors and transducers, electronic devices, magnetic storage, electromagnetic and microwave absorption, and magnetic actuators.

## Figures and Tables

**Table 1:** Summary of polymer nanocomposites with their corresponding properties.

Application	Polymer Matrix	Magnetic Filler	Properties	References
Supercapacitors	PANI	Fe_3_O_4_	High specific capacitance Cycling stability Excellent capacitance retention	[130]
		BaFe_12_O_19_	Specific capacitance (225–330 F/g)	[131]
		Cr_2_O_3_-graphene oxide	A high specific capacitance value of 525 F/g	[132]
		SnO_2_	A specific capacitance of 337 F/g	[134]
		HY zeolite/SnO_2_	A maximum capacitance of 1085 F/g	[135]
		CN/SiO_2_	A specific capacitance value of 221 F/g	[136]
	PPy	Cr_2_O_3_-graphene oxide	A high specific capacitance value of 495 F/g	[132]
	PEDOT	rGO/TiO_2_	Improved specific capacitance depending on the ratio content	[139]
		rGO/MnFe_2_O_4_	A good specific capacitance of 298.97 F/g	[140]
Sensors	PANI	ZnO	A magnetic flux of 0.5 T Resistance of 865 k Response time of 18.2 s Recovery time of 5.1 s	[145]
		CuFe_2_O_4_	Fast response time	[146]
	PTh	ZrO_2_	High thermal stability Highest electrical conductivity	[147]
	Polydopamine	Fe_3_O_4_	Enhanced limit of detection of DDT	[148]
	PDA	Clay/Fe_3_O_4_	Improved magnetic saturation Highest detection of diazinon Enhanced adsorption capacity	[149]
LED	MEH-PPV	Co_x_Zn_1−x_O	Band gap narrowing Luminescence quenching	[153]
		Fe_x_Zn_1−x_O	Enhanced electroluminescence	[154]
	Poly(*p*-phenylenediamine	ZnO, Fe_3_O_4_, or TiO_2_	Improved soft magnetic properties Semiconducting material	[155]
	PEDOT: PSS	CDs/ZnS	Decreased turn-on voltage	[156]
	PSEBS	CsPbX_3_ (X = Cl/Br, Br, Br/I)	Flexible films Wide color gamut	[157]
	PMMA	FAPbBr_3_ QDs	Enhanced Lumen Efficiency Improved Color Rendering Index	[159]
Solar cells	π-conjugated polymer donor	Nanoscale coating	Improved stability and efficiency	[160,162]
	P3HT	Fe_3_O_4_	Improved magnetic responsiveness Controlled and stable morphologies	[163]

## Data Availability

Not applicable.
